# Psychosocial Effects of Fractured Anterior Teeth among Rural Children

**DOI:** 10.5005/jp-journals-10005-1348

**Published:** 2016-06-15

**Authors:** Ramesh Venkatesan, Mohan Naveen, Ravi Teja, Shankar Paulindraraj, Sai K Vallabhaneni, Selva B Arumugam

**Affiliations:** 1 Senior Lecturer, Department of Pediatric and Preventive Dentistry, Indira Gandhi Institute of Dental Sciences, Sri Balaji Vidyapeeth University, Puducherry, India; 2 Pediatric Dentist, Private Practice, Villupuram, Tamil Nadu, India; 3 Senior Lecturer, Department of Oral Pathology and Microbiology, SIBAR Institute of Dental Sciences, Guntur, Andhra Pradesh, India; 4 Senior Lecturer, Department of Pediatric and Preventive Dentistry, Madha Dental College and Hospital, Chennai, Tamil Nadu, India; 5 Senior Lecturer, Department of Pediatric and Preventive Dentistry, SIBAR Institute of Dental Sciences, Guntur, Andhra Pradesh, India; 6 Senior Lecturer, Department of Pediatric and Preventive Dentistry, Indira Gandhi Institute of Dental Sciences, Sri Balaji Vidyapeeth University, Puducherry, India

**Keywords:** Attribute, Fracture, Psychosocial, Trauma.

## Abstract

**Aim:** The aim of the study is to determine how rural children view children with visible incisor fracture.

**Materials and methods:** Class 7 (aged 11-12 years) and class 10 (aged 14-15 years) schoolchildren (the participants) were invited to make a social judgment about the color photograph of two children (the subjects). Participants were randomly allocated either (i) pictures of children without incisor fracture or (ii) pictures of the same children whose photographs had been digitally modified to visible incisor fracture. Using a child-centered questionnaire, participants rated subjects using a four-point Likert scale for three negative and six positive attributes. Total attribute scores were tested for significant differences, according to whether the subject had visible incisor fracture or not, using multivariate analysis of variance (p < 0.05).

**Results:** Both class 7 and 10 children rated subjects with visible incisor fracture more negatively than the subjects without incisor fracture. Female participants of class 10 have rated the male subject with incisor fracture significantly negatively (p < 0.01) than male subject without incisor fracture.

**How to cite this article:** Venkatesan R, Naveen M, Teja R, Paulindraraj S, Vallabhaneni SK, Arumugam SB. Psychosocial Effects of Fractured Anterior Teeth among Rural Children. Int J Clin Pediatr Dent 2016;9(2):128-130.

## INTRODUCTION

Increase in concern toward dental appearance can be observed among schoolchildren.^1,2^ Schoolchildren can be unkind and hostile to those with visible differences, with teasing and bullying being everyday occurrences.^3^ A total of 75% of children cited teasing or bullying about their appearances as causing considerable distress.^4^ This may not pertain only to children in urban areas. When self-esteem of children in rural towns was assessed, it was found that rural children’s self-perceptions are not distinctly different from urban and suburban children.^5^ Teeth holds a greater share of attention in facial esthetics, and a dental appearance that deviates from acceptable norms may even negatively affect an individual.^6^ Thus, the aim of our study is to determine the psychosocial effects of fractured anteriors among rural children and to explore the effect of age and gender on character perception by rural children.

## MATERIALS AND METHODS

### The Photographs

**Figs 1A and B:**
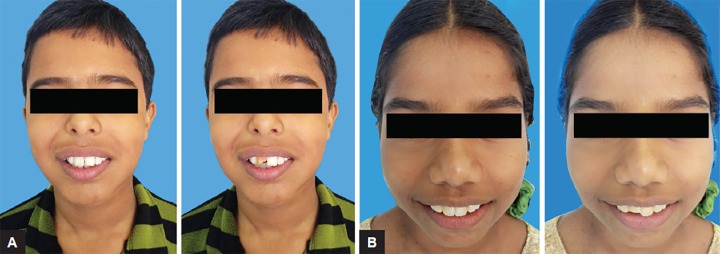
Full face photographs of the two subjects in this study: (A) without incisor fracture and (B) with fractured incisor (digitally modified)

Full face color photographs (Figs 1A and B) of a boy and a girl, aged between 11 and 15 years, were taken after obtaining informed consent from the parents. Copies of the same photographic images were digitally modified so that it appeared like the incisor was either fractured or fractured and discolored.

### The Questionnaire

The questionnaires used in this study were originally developed by Rodd et al^7^ and were found to have good internal consistency. The questionnaire included nine descriptors (six positive and three negative attributes) within three different domains: Social competence, psychosocial adjustment, and intellectual competence. The positive descriptors included were clever, kind, honest, confident, careful, and helpful and the negative descriptors were rude, stupid, and naughty. Since English is not the mother tongue of the participants, the descriptor words were translated in regional language―Tamil.

Study participants included all class 7 and class 10 pupils at a secondary school at Annamalai Nagar, Chidambaram, India. The school administrator randomly allocated the questionnaires and photographs to each class, so that half of the class 7 and 10 groups received the trauma photographs and the remaining classes received the nontrauma photographs. They were asked to rate each child for the nine social attributes. A four=point Likert scale was used to record responses, ranging from “strongly agree,” “agree,” and “disagree” to “strongly disagree.” The participants were not told that the study was dentally related. Participants were not allowed to confer during completion of their questionnaire.

### Data Analysis

The total attribute score was calculated by summing the response codes. The positive attributes namely clever, kind, honest, confident, careful, and helpful were coded as “strongly agree” = 4; “agree” = 3; “disagree” = 2; “strongly disagree” = 1. The negative attributes of rude, stupid, and naughty had the scoring reversed. Thus, a high score (maximum of 36) would correlate with positively judged subject and a low score (minimum of 9) would correlate with a negatively viewed subject. The use of parametric tests has been reported as appropriate for analysis of similarly obtained social attribute scales in previous studies.^8,9^ The two dependent variables were the mean total attribute score for each of the paired photographs. The fixed factors were gender of the participant (male or female), the school class (class 7 or 10), and incisor status (presence or absence of visible incisor fracture). Significant results from multivariate analysis of variance were examined using descriptive statistics and *post hoc* multiple comparisons. The significance level was set at p < 0.05.

## RESULTS

The participants of the study were class 7 and 10 students of a rural school. The total number of participants was 199, of whom 111 (55.8%) students belonged to class 7 and 88 (44.2%) students were from class 10. Number of male participants in class 7 was 79 and 32 were female. And there were 53 male and 35 female participants in class 10.

Table 1 provides the mean attribute scores for the subject of each photograph (with or without incisor fracture) according to the age and gender of the participant. Both class 7 and 10 participants rated subject with incisor fracture more negatively than subject without incisor fracture for both male and female subjects. Female participants of class 10 have rated the male subject with incisor fracture significantly negatively (p < 0.01) than male subject without incisor fracture. Male participants in total have rated female subject with incisor fracture more negatively (p = 0.01) than the female participants. None of the remaining main effects or interactions was statistically significant.

**Table 1:** **Table 1:** Mean (standard deviation) attribution scores for children with and without visible incisor trauma according to the age and gender of the participant

*Photographic subject*		*Class 7 total* *(n = 111)*		*Class 7 boys* *(n = 79)*		*Class 7 girls* *(n = 32)*		*Class 10 total* *(n = 88)*		*Class 10 boys* *(n = 53)*		*Class 10 girls* *(n = 35)*	
Subject 1 (male) with incisor #		25.79 (3.59)		25.40 (3.89)		25.70 (4.09)		25.45 (4.48)		25.00 (4.84)		24.83 (4.03)	
Subject 1 (male) without incisor #		26.78 (4.18)		27.27 (4.22)		26.75 (2.71)		25.96 (3.52)		25.29 (4.03)		27.00 (2.40)	
Subject 2 (female) with incisor #		24.08 (5.11)		22.11 (3.34)		25.50 (6.57)		24.96 (5.09)		23.53 (4.93)		27.33 (4.66)	
Subject 2 (female) without incisor #		24.55 (4.22)		23.58 (4.30)		27.00 (4.73)		26.25 (4.22)		24.83 (4.69)		28.44 (2.13)	

## DISCUSSION

The aim of this study was to determine whether children with visible incisor fracture are viewed more negatively than those with intact incisors by rural children. There is considerable evidence to suggest that teeth hold a greater share of attention in the face. According to Newton et al,^8^ in the absence of other information, the judgments an individual makes concerning the personal characteristics of others are influenced by dental appearance. Feng et al^9^ reported that subjects with less dental disease were judged to be better adjusted and more intellectually competent.

In the present study, there was no marked difference in the evaluation of any of the subjects according to age. Though not statistically significant, both boys and girls of class 7 rated both the subjects with incisor fracture more negatively than subjects without incisor fracture. The mean attribute score given by class 10 boys toward both male and female subjects with incisor fracture was consistently negative than subjects without incisor fracture, but were not statistically significant, whereas the mean attribute score given by class 10 girls toward the male subject with incisor fracture was significantly negative than male subject without incisor fracture. In a similar study, Rodd et al^7^ found that children aged 11 to 12 years would make negative social judgment on the basis of poor dental appearance. Kershaw et al^10^ reported that decayed dental appearance led to more negative judgments over the four personality categories (social competence, intellectual ability, psychological adjustment, and relationship satisfaction). Whitened teeth led to more positive appraisals.

Since there is remarkable decline in prevalence and severity of dental caries, traumatic dental injury has become the most serious dental public health problem in children.^11^ Many studies state that prevalence of traumatic dental injuries ranges from 4 to 19.5%, of which the percentage of traumatized teeth that had undergone treatment is very low (3.37%).^12-14^ This low percentage is due to low level of awareness among the parents of the children in the rural areas.^14^

## CONCLUSION

From the results of this study, it is observed that both 11 to 12-year-old children and 14- to 15-year-old children attribute negative personality characteristics to other children with incisor fracture. It is evident that social judgments made by rural children toward peer-aged children with incisor fracture may have adverse psychosocial effects. So efforts should be made to increase knowledge among the rural children and their parents about the fracture to anterior teeth and its psychosocial effects.
